# Peripheral Giant Cell Granuloma in a Child Following Tooth Extraction

**DOI:** 10.5334/jbsr.3819

**Published:** 2025-03-04

**Authors:** Hafsa Selmani, Michaela Kubincova, Yannick De Brucker

**Affiliations:** 1 Department of Radiology, University Hospital Brussels, Belgium; 2 Department of Radiology, University Hospital Brussels; Radiologie Buggenhout, Buggenhout, Belgium

**Keywords:** Peripheral giant cell granuloma, tooth extraction, CECT, orthopantomogram

## Abstract

*Teaching point:* Peripheral giant cell granuloma (PGCG) is a common benign hyperplastic reactive lesion that originates from the soft tissues of the oral cavity and should be considered following a tooth extraction.

## Case History

**Figure 1:**
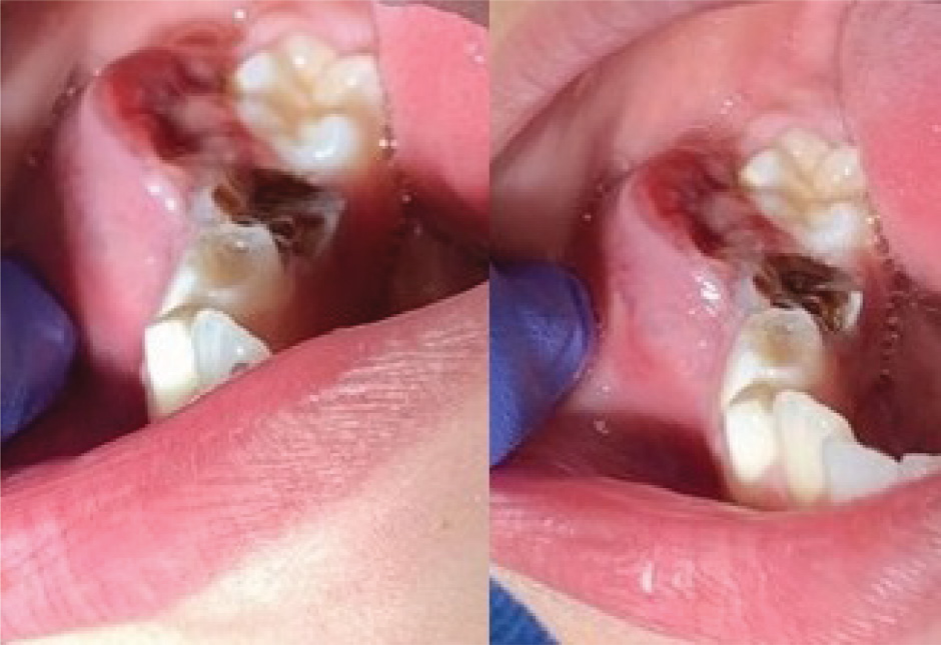
Intraoral view of a reddish sessile lesion in the right mandibular region.

**Figure 2:**
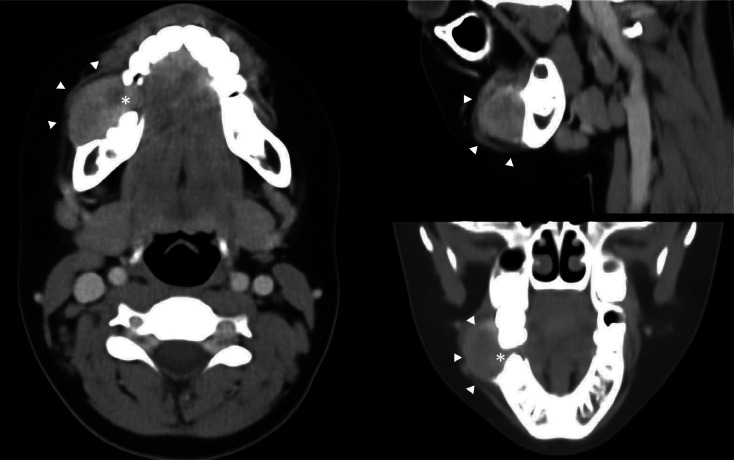
CECT in soft tissue window showing a homogeneously enhancing solid gingival lesion at the buccal aspect (asterisk) causing bulging of the buccinator muscle (white arrowheads).

**Figure 3:**
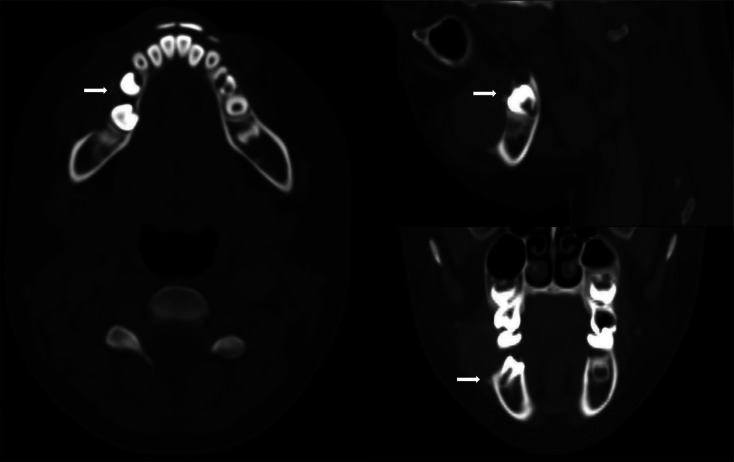
CECT in bone window revealing superficial bone erosions at outer alveolar ridge (white arrows).

An 8‑year‑old boy with a history of primary second molar extraction in the right mandible presented to the department of maxillofacial surgery with a painful swelling in the right lower jaw. Clinical examination revealed a reddish sessile lesion with an erythematous and ulcerated surface (Figure 1). Contrast‑enhanced computed tomography (CECT) of the right mandibular region demonstrated a non‑calcified, homogeneously enhancing solid lesion, measuring approximately 2 cm, arising from the gingiva at the outer alveolar margin (Figure 2, asterisk) and causing bulging of the buccinator muscle (Figure 2, arrowheads). Bone window reconstructions revealed superficial bone erosion (Figure 3, arrows). Histopathological examination following surgical removal confirmed the diagnosis of peripheral giant cell granuloma (PGCG).

## Comments

PGCG is a benign hyperplastic reactive lesion that arises from the periosteum or periodontal membrane of the oral cavity, typically following local irritation or chronic trauma. PGCG accounts for approximately 7% of all benign tumors of the jaw, predominantly affecting the lower jaw and the premolar or molar regions. While there is a higher prevalence of PGCG among middle‑aged and elderly patients, it is more commonly seen in boys than in girls during childhood [1]. The lesion may exhibit more aggressive behavior in children, with a higher likelihood of recurrence. Clinically, PGCG presents as a reddish to bluish soft tissue lesion that can be sessile or pedunculated, and may be ulcerated and prone to bleeding. An orthopantomogram (OPG) is often used for initial evaluation, but it is insufficient on its own for a thorough assessment of soft tissues.

Computed tomography (CT) imaging and OPG typically reveal superficial destruction of the alveolar margin or crest of the interdental bone, characterized by superficial bone erosion (referred to as saucerization or cuffing), newly formed vertical osteoid spicules at the base of the lesion, and widening of the adjacent periodontal space. Cuffing has been reported in nearly one‑third of cases.

CECT is utilized as a primary diagnostic tool to avoid invasive procedures and to assess the origin of the mass. However, a biopsy followed by histopathological examination is essential for establishing a definitive diagnosis. Unlike central giant cell granuloma (CGCG), there is limited literature regarding the imaging characteristics of PGCG on magnetic resonance imaging (MRI). In cases of acute onset, CT is generally sufficient for initial evaluation, and diagnosis can often be made without further MRI workup. The differential diagnosis of PGCG includes pyogenic granuloma, peripheral ossifying fibroma, and gingival peripheral fibroma. The mainstay treatment is excision and curettage, along with maintaining proper oral hygiene to prevent recurrence.
